# Comprehensive Analysis of Long Non-coding RNA and mRNA Transcriptomes Related to Hypoxia Adaptation in Tibetan Sheep

**DOI:** 10.3389/fvets.2021.801278

**Published:** 2022-01-24

**Authors:** Zengkui Lu, Chao Yuan, Jianye Li, Tingting Guo, Yaojing Yue, Chune Niu, Jianbin Liu, Bohui Yang

**Affiliations:** ^1^Lanzhou Institute of Husbandry and Pharmaceutical Sciences, Chinese Academy of Agricultural Sciences, Lanzhou, China; ^2^Sheep Breeding Engineering Technology Research Center of Chinese Academy of Agricultural Sciences, Lanzhou, China

**Keywords:** Tibetan sheep, lung, hypoxic adaptation, long non-coding RNAs, transcriptome

## Abstract

Tibetan sheep have lived on the Qinghai-Tibet Plateau for a long time, and after long-term natural selection, they have shown stable genetic adaptability to high-altitude environments. However, little is known about the molecular mechanisms of the long non-coding (lnc)RNAs involved in the adaptation of Tibetan sheep to hypoxia. Here, we collected lung tissues from high-altitude Tibetan sheep and low-altitude Hu sheep for RNA sequencing to study the regulatory mechanisms of the lncRNAs and mRNAs in the adaptation of Tibetan sheep to hypoxia. We identified 254 differentially expressed lncRNAs and 1,502 differentially expressed mRNAs. We found 20 pairs of cis-regulatory relationships between 15 differentially expressed lncRNAs and 14 protein-coding genes and two pairs of trans-regulatory relationships between two differentially expressed lncRNAs and two protein-coding genes. These differentially expressed mRNAs and lncRNA target genes were mainly enriched in pathways related to lipid metabolism and immune function. Interaction network analysis showed that 17 differentially expressed lncRNAs and 15 differentially expressed mRNAs had an interactive relationship. Additionally, we used six differentially expressed lncRNAs and mRNAs to verify the accuracy of the sequencing data via qRT-PCR. Our results provide a comprehensive overview of the expression patterns of the lncRNAs and mRNAs involved in the adaptation of Tibetan sheep to hypoxia, laying a foundation for further analysis of the adaptations of plateau animals.

## Introduction

The Qinghai-Tibet Plateau is the highest plateau worldwide. It is known as the “Third Pole” of the earth and is known for its low oxygen, low temperatures, and strong ultraviolet radiation. Along with human migration and settlement, many domestic animals have reproduced for generations in these harsh living conditions and have thus adapted unique and distinctive characteristics at the morphological, physiological and genetic levels (1–3). Scientists have used large-scale omics data to reveal the adaptive genetic mechanisms of domestic animals on the Qinghai-Tibet Plateau and identified a number of plateau-adaptive candidate genes, including *EPAS1* (endothelial PAS domain-containing protein 1), *EGLN1* (Egl-9 homolog 1) and *VEGF* (vascular endothelial growth factor), in the HIF (hypoxia inducible factor) hypoxia-inducing pathway (4–7).

Long non-coding RNA (lncRNA) is non-coding RNA that contains >200 nucleotides. LncRNA has important roles in many life activities such as the dosage compensation effect, epigenetic regulation, cell cycle regulation and cell differentiation regulation (8, 9). Studies have shown that lncRNA can bind to HIF-1α and activate its expression, thereby playing an important role in hypoxia-induced tumor cells (10–14). LncRNA may also play an important role in the adaptability of domestic animals to plateaus. Recent studies have shown that lncRNA is involved in the adaptability of Tibetan chickens and yaks to plateaus; however, the regulatory mechanisms of this lncRNA remain largely unknown (15, 16).

To reveal the potential role of lncRNA in the adaptability of domestic animals to plateaus, we selected sheep from different altitudes and collected lung tissue for transcriptomic sequencing. We used an integrated analysis of mRNA and lncRNA data to explore the molecular mechanisms of Tibetan sheep's adaptability to plateaus, find the genes and regulatory pathways related to this adaptability, and provide a theoretical basis for Tibetan sheep production and breeding.

## Materials and Methods

### Sample Collection

We collected lung tissues from six healthy 18-month-old rams, of which, three were from the Qinghai-Tibet Plateau (Zashijia sheep, Qumalai County, Yushu Tibetan Autonomous Prefecture, Qinghai, China, altitude ~4,800 m), and three were from the plains of China (Hu sheep, Minqin County, Wuwei, Gansu, China, altitude ~1,400 m). All six sheep grazed and received supplemented feed and were euthanized by an intravenous injection of phenobarbital solution (Fatal-Plus, 10 mg/kg body weight, Vortech Pharmaceuticals, MI, USA). Tissues were collected from the middle lobe of the right lung and stored in liquid nitrogen, then used for RNA extraction.

### RNA Extraction, Library Construction, and Sequencing

We used TRlzol reagent (Invitrogen, Carlsbad, CA, USA) to extract the total RNA and remove rRNA per the manufacturer's instructions. RNA samples that qualified through quality inspection were used for library construction in accordance with the instructions provided by Illumina (Illumina, CA, USA). The RNA was randomly interrupted into short 200–500-nt fragments, and the first cDNA strand was synthesized using random primers. Buffer, dNTPs (dUTP instead of dTTP), RNase H and DNA polymerase I were added to synthesize the second cDNA strand. The cDNA was purified using the QiaQuick PCR kit, then EB buffer was added to elute the proteins, the ends were repaired, and base A and sequencing adapters were added. Uracil-N-glycosylase was used to degrade the second chain. Agarose gel electrophoresis was used to select the appropriate fragments for PCR amplification. Qualified libraries were sequenced using the HiSeq™ 4000 platform.

### Raw Data Filtering, Comparison, and Splicing

After sequencing, the raw data (raw reads) were obtained, and fastp software was used for quality control to obtain clean reads. Quality control was performed by removing reads containing adapters, reads with an N content > 10%, reads with only A bases, and low-quality reads (the number of bases with a quality value of Q ≤ 20 accounted for the total number of reads above 50). We used Bowtie2 software to compare clean reads to the rRNA database and remove the ribosomal reads. We used HISAT2 software to compare the clean reads to the sheep reference genome (Oar_v4.0), set the parameter to -rna-strandness RF, and set the remaining parameters to the default values. We used Stringtie software to assemble the clean reads compared to the sheep reference genome and used Cuffmerge software to merge each transcript. We used Cuffcompare software to compare the assembled transcripts with known transcript types (NCBI Refseq, Ensembl transcripts, UCSC), identify known mRNAs, and screen new non-coding transcripts (novel ncRNA).

### lncRNA Identification and Target Gene Prediction

To screen for new non-coding transcripts (novel ncRNA), we filtered transcripts with ≥200 bp and ≥2 exons. We used CPC2, CNCI and Pfam software to predict the coding ability of the new transcripts and took the intersection of the transcripts without coding potential as candidate lncRNAs. The lncRNA target genes included cis-target and trans-target genes. mRNA 50 kb upstream and downstream of the lncRNA were used as the cis-target, and mRNA whose expression correlation coefficient was >0.9 was used as the trans-target.

### Identification of Differentially Expressed mRNAs and lncRNAs

For the FPKM value, we used StringTie software to calculate the mRNA and lncRNA expression levels, with the equation FPKM = total fragments/mapped reads (millions) × exon length (kB). We used DESeq2 software to analyze the differences between the mRNA and lncRNA, standardize the reads counts, calculate the hypothesis test probability (*p*-value), and perform multiple hypothesis test corrections to obtain the false discovery rate (FDR). From the difference analysis, *P* < 0.05 and a fold change > 1.5 were used to screen significantly differentially expressed mRNA and lncRNA.

### Functional Enrichment Analysis of Differentially Expressed Genes

We mapped the differential genes to each term in the gene ontology (GO) database (http://www.geneontology.org/), calculated the number of differential genes in each term, and obtained a list of differential genes with specific GO functions. The hypergeometric distribution test was used to calculate the *P*-value for the significantly enriched GO functions, then the *P*-value was corrected using the Benjamini-Hochberg multiple test (FDR). GO items with FDR ≤ 0.05 were considered significantly enriched. We used the Kyoto Encyclopedia of Genes and Genomes (KEGG) database to annotate and classify the differentially expressed genes with pathway functions. The KEGG pathway functional enrichment method is similar to the GO functional enrichment analysis.

### Construction of the lncRNA-mRNA Network

We used the interaction relationship in the STRING protein interaction database (http://string-db.org) to analyze the differential gene interaction network. The differential gene set was extracted from the database, and the interaction network diagram was constructed and visualized using Cytoscape software.

### Validation of lncRNA and mRNA Expression by qRT-PCR

To test the accuracy of the sequencing results, we selected six differentially expressed mRNA and lncRNA for qRT-PCR verification. We used a cDNA synthesis kit to reverse transcribe the extracted total RNA into cDNA. We used Oligo7 software to design primers and perform specific detections in NCBI. We used the TransStart Green qPCR SuperMix and LightCycler 480 II instruments to perform qRT-PCR and ran each sample in triplicate to ensure the accuracy of the quantitative results. The 2^−ΔΔCt^ method was used to calculate the relative expression of the target genes, and *ACTB* (beta-actin) was used as the internal reference gene. Supplementary Table 1 lists the primers used in this study.

### Statistical Analysis

All data are presented as the mean ± standard deviation and were analyzed using Student's t-test in SPSS software. *p* < 0.05 was considered statistically significant.

## Results

### Overview of the Sequencing Data

Using the Illumina HiSeq™ 4000 platform, the six constructed libraries were paired-end sequenced. Table 1 shows the data quality control and genome comparison results. The six libraries produced 499,954,984 raw reads; 498,979,644 clean reads remained after quality control. The average rate of the clean reads was 99.81%. The GC content of the six samples was 47.57–49.25%, which was consistent with the base composition law of Q20 ≥ 97.63% and Q30 ≥ 93.17%. We used HISAT2 software to compare the clean reads to the sheep reference genome. Approximately 92.47% of the reads could be accurately compared, and the match rate was high. Only data aligned to the sheep reference genome were used for further bioinformatics analysis.

**Table 1:** Summary of sequenced RNA-seq data.

**Items**	**HS1**	**HS2**	**HS3**	**TS1**	**TS2**	**TS3**
Raw datas	81,317,688	81,303,748	81,256,760	79,261,536	88,127,470	88,687,782
Clean datas	81,181,820 (99.83%)	81,140,622 (99.80%)	81,085,206 (99.79%)	79,104,972 (99.80%)	87,948,784 (99.80%)	88,518,240 (99.81%)
GC content	49.25%	47.57%	47.58%	47.96%	48.22%	47.74%
Q20 (%)	97.87%	97.68%	97.63%	98.56%	98.55%	98.57%
Q30 (%)	93.63%	93.29%	93.17%	95.38%	95.37%	95.40%
Multiple mapped	2,698,512 (3.32%)	2,282,439 (2.81%)	2,239,924 (2.76%)	2,357,259 (2.98%)	2,885,038 (3.28%)	2,691,472 (3.04%)
Unique mapped	72,640,831 (89.50%)	72,720,281 (89.65%)	72,944,114 (90.01%)	71,800,901 (90.80%)	79,348,585 (90.26%)	80,450,762 (90.92%)
Unmapped	5,820,851 (7.17%)	6,108,764 (7.53%)	5,856,384 (7.23%)	4,917,942 (6.22%)	5,678,065 (6.46%)	5,344,200 (6.04%)
Total mapped	75,339,343 (92.83%)	75,002,720 (92.47%)	75,184,038 (92.77%)	74,158,160 (93.78%)	82,233,623 (93.54%)	83,142,234 (93.96%)

### lncRNA and mRNA Feature Analysis

**Figure 1:**
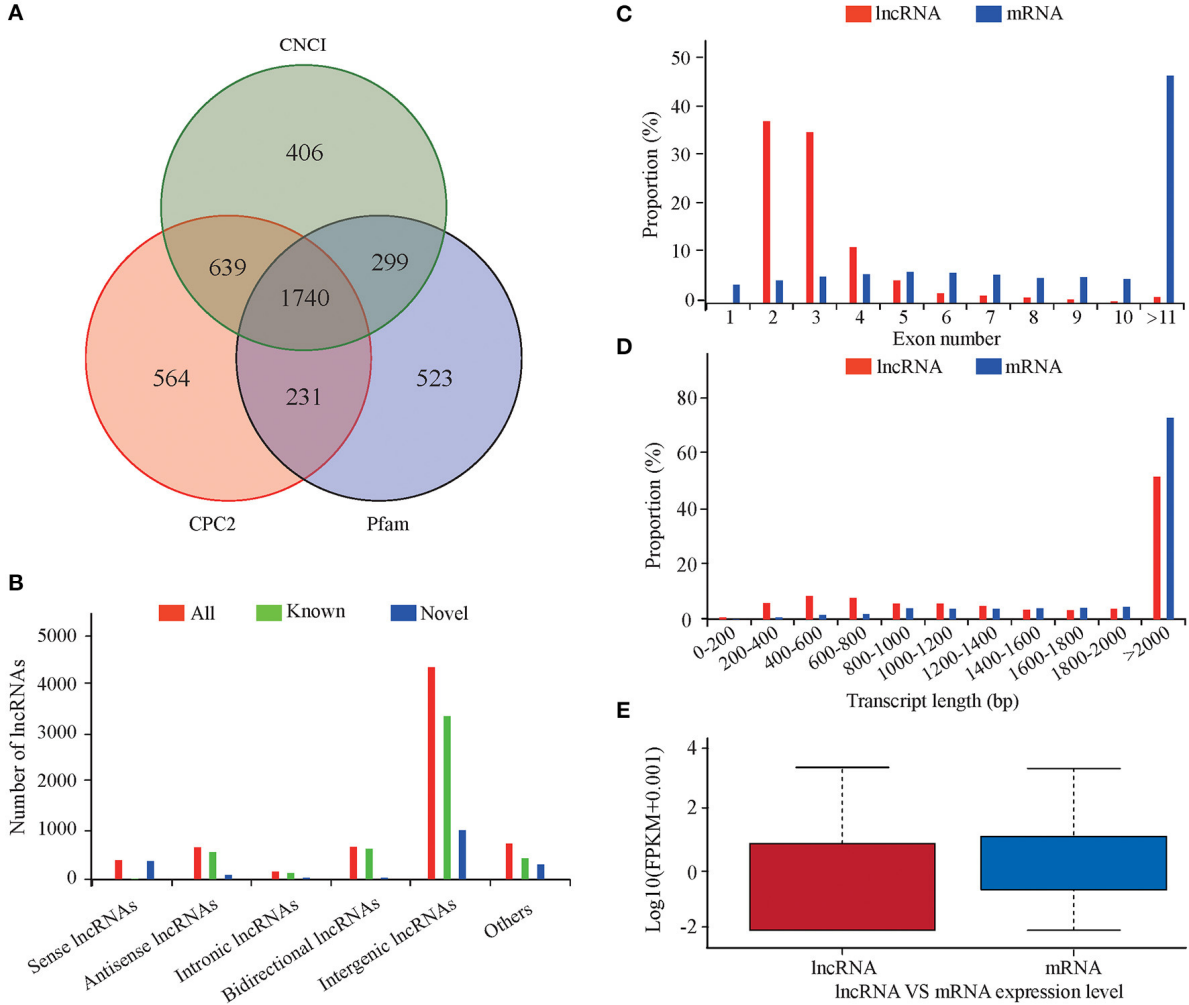
Characteristics of lncRNAs and mRNAs in the lung tissues of sheep at different altitudes. **(A)** Screening of candidate lncRNAs by CNCI and CPC2. **(B)** Data on lncRNA transcript types. **(C)** Distribution of exon numbers in lncRNAs and mRNAs. **(D)** Length distribution of lncRNAs and mRNAs. **(E)** Expression levels of lncRNAs and mRNAs.

An average of 17,904 expressed genes and 5,615 lncRNAs were identified from the six libraries. Using CNCI, CPC2, and Pfam to predict the coding ability of the new transcripts yielded 1,740 new lncRNAs (Figure 1A), including 357 sense, 89 antisense, 31 intronic, 37 bidirectional, 941 intergenic, and 285 other lncRNAs (Figure 1B). Most lncRNAs had two or three exons, which was significantly less than the number of exons in the mRNA (Figure 1C). The length distribution range of the lncRNA and mRNA was basically the same, but the lncRNA was longer than the mRNA (Figure 1D). The lncRNA expression level was lower than that of the mRNA (Figure 1E).

### Identification and Analysis of Differentially Expressed lncRNA and mRNA

**Figure 2:**
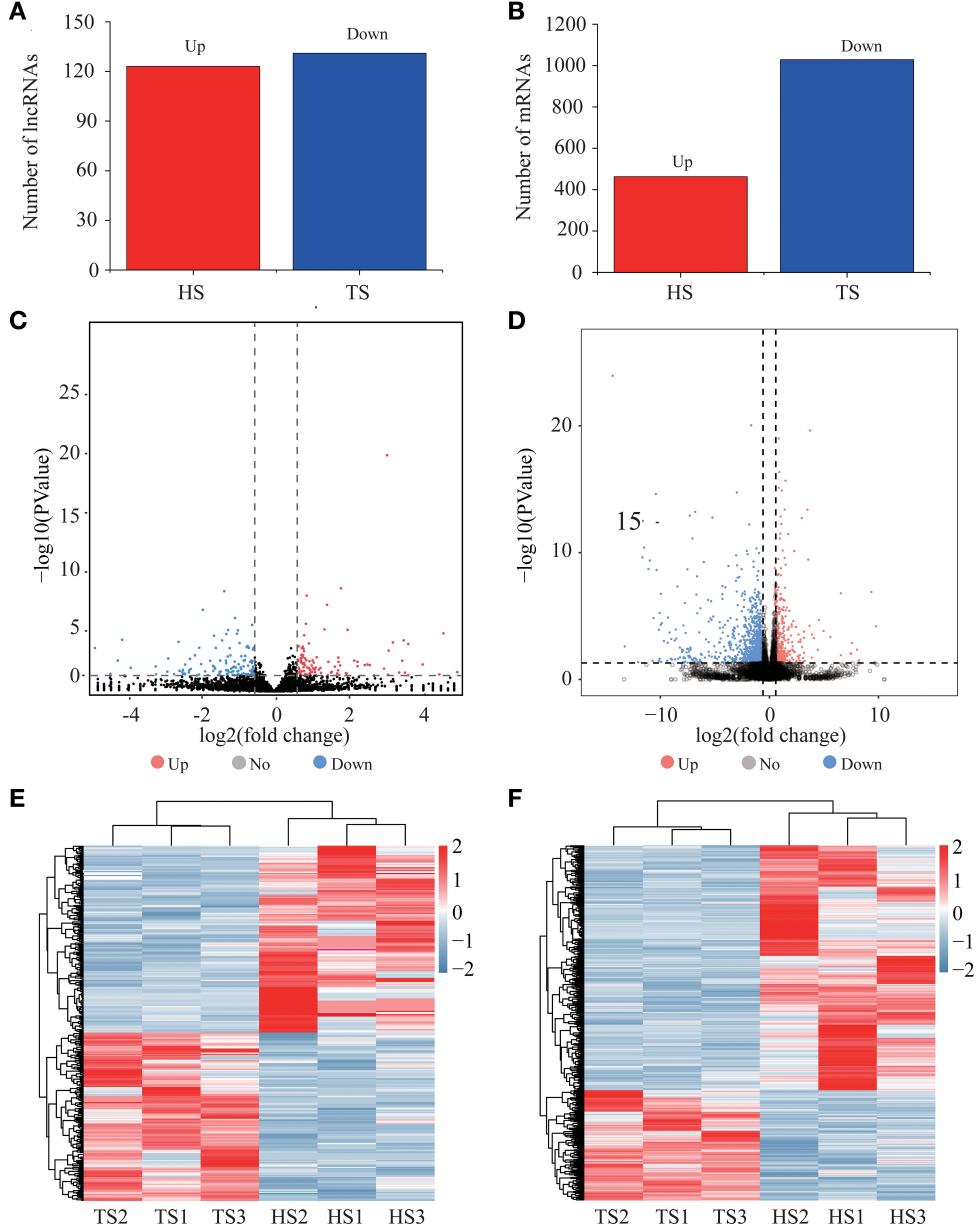
Expression analysis of lncRNAs and mRNAs. **(A)** Numbers of upregulated and downregulated differentially expressed lncRNAs. **(B)** Numbers of upregulated and downregulated differentially expressed mRNAs. **(C)** Volcano plots displaying differentially expressed lncRNAs. **(D)** Volcano plots displaying differentially expressed mRNAs. **(E)** Heatmap of differentially expressed lncRNAs. **(F)** Heatmap of differentially expressed mRNAs.

We identified 254 differentially expressed lncRNAs between the Tibetan and Hu sheep, of which, 123 were upregulated, and 131 were downregulated (Figures 2A,C; Supplementary Table 2). The five lncRNAs with the most significant differential expression were MSTRG.19949.1, MSTRG.6422.3, MSTRG.6796.1, MSTRG.16435.3, and MSTRG.19804.1. We identified 1502 differentially expressed mRNAs between the Tibetan and Hu sheep, of which 469 were upregulated and 1,033 were downregulated (Figures 2B,D; Supplementary Table 3). The five most significantly differentially expressed mRNAs were *NKIRAS2* (NK-κB inhibitor-interacting Ras-like 2), *SEPW1* (selenoprotein W), *PDZK1* (PDZ domain-containing 1), *PEAK1* (pseudopodium enriched atypical kinase 1), and *KIAA1549*. Among these, *NKIRAS2* is involved in immune response, *SEPW1* is involved in oxidative stress, and *PDZK1* is involved in fat metabolism. Additionally, the differentially expressed lncRNA and mRNA were combined into one group, and the differentially upregulated and downregulated lncRNA and mRNA were combined into one group (Figures 2E,F). The differentially expressed lncRNA and mRNA had good reproducibility in the groups.

### Prediction of lncRNA Target Genes and Analysis of lncRNA-mRNA Interaction

The 254 differentially expressed lncRNAs were predicted for cis- and trans-target genes (Table 2). We found 20 pairs of cis-regulatory relationships between 15 differentially expressed lncRNAs and 14 protein-coding genes, of which, three lncRNAs were upstream of the target gene, and 17 lncRNAs were downstream. There were also two pairs of trans-regulatory relationships between the two differentially expressed lncRNAs and the two protein-coding genes. Among these target genes, *SH2D7* (SH2 domain containing) and *IL12A* (interleukin-12 A) are involved in immune response, *LPAR1* (lysophosphatidic acid receptor 1) and *FKBP10* (FK506 binding protein 10) are involved in signal transduction, *STEAP4* (six-transmembrane epithelial antigen of prostate 4) is involved in oxidative stress, *KCNG3* (voltage-gated channel subfamily G member 3) is involved in ion transport, and *PHACTR1* (phosphatase and actin regulator 1) is involved in pulse pressure regulation.

**Table 2:** Differentially expressed lncRNAs and their targeted mRNAs.

**Type**	**lncRNA ID**	**chr**	**Gene ID**	**Up/down stream**	**Symbol**
cis	XR_001026700.1	NC_019459.2	ncbi_443346	Upstream	LPAR1
	MSTRG.2514.1	NC_019459.2	MSTRG.2515	Downstream	–
	MSTRG.4734.2	NC_019460.2	ncbi_101115443	Downstream	KCNG3
	MSTRG.5378.1	NC_019460.2	ncbi_101119634	Downstream	SLC26A10
	MSTRG.6522.1	NC_019461.2	ncbi_101111187	Downstream	STEAP4
	MSTRG.10206.1	NC_019466.2	MSTRG.10204	Downstream	–
	MSTRG.10206.1	NC_019466.2	MSTRG.10208	Downstream	–
	XR_001435094.1	NC_019466.2	MSTRG.10204	Downstream	–
	XR_001435094.1	NC_019466.2	MSTRG.10208	Downstream	–
	MSTRG.10206.3	NC_019466.2	MSTRG.10204	Downstream	–
	MSTRG.10206.3	NC_019466.2	MSTRG.10208	Downstream	–
	MSTRG.10206.4	NC_019466.2	MSTRG.10204	Downstream	–
	MSTRG.10206.4	NC_019466.2	MSTRG.10208	Downstream	–
	MSTRG.10898.1	NC_019467.2	ncbi_101107613	Upstream	LOC101107613
	XR_001435802.1	NC_019472.2	ncbi_101118897	Downstream	LOC101118897
	MSTRG.16852.1	NC_019475.2	ncbi_101114647	Downstream	SH2D7
	XR_001022162.2	NC_019475.2	ncbi_105603299	Upstream	LOC105603299
	XR_001022162.2	NC_019475.2	ncbi_105601883	Downstream	LOC105601883
	MSTRG.17316.1	NC_019476.2	ncbi_101111131	Downstream	EXOG
	MSTRG.18414.1	NC_019477.2	ncbi_101115323	Downstream	PHACTR1
antisence	MSTRG.11948.1	NC_019468.2	ncbi 101108933	DOWNSTREAM	FKBP10
	MSTRG.1821.1	NC_019458.2	ncbi 443064	DOWNSTREAM	IL12A

**Figure 3:**
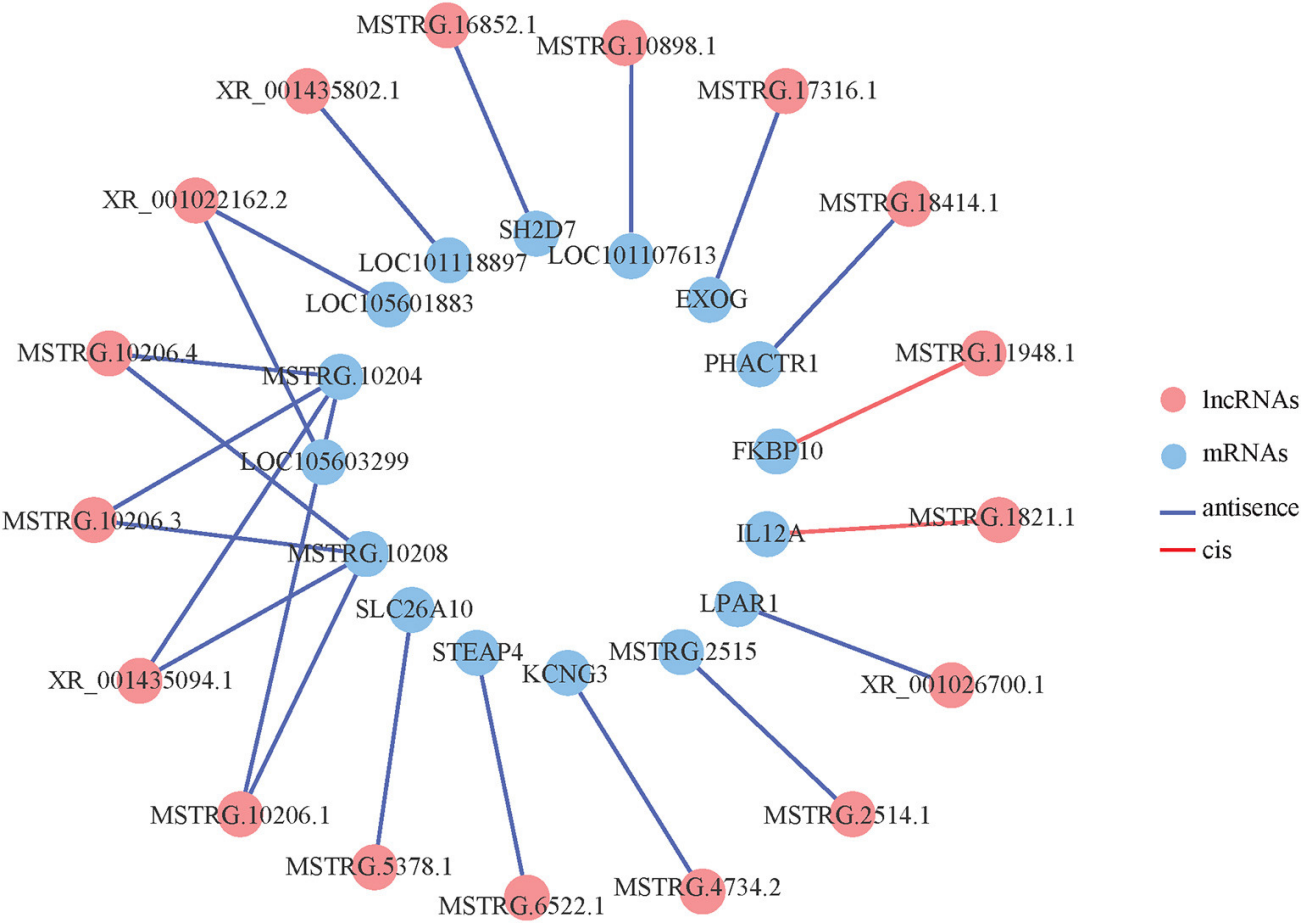
Co-expression network of differentially expressed lncRNAs and differentially expressed mRNAs.

We constructed an interaction network based on these differentially expressed lncRNAs and mRNAs, and 17 differentially expressed lncRNAs and 15 differentially expressed mRNAs had an interaction relationship (Figure 3). Among the differentially expressed lncRNAs, MSTRG.10206.4, MSTRG.10206.3, XR_001435094.1, MSTRG.10206.1 and XR_001022162.2 interacted with two mRNAs, and the rest of the lncRNAs interacted with only one mRNA. Among the differentially expressed mRNAs, MSTRG.10204 interacted with four lncRNAs, MSTRG.10208 interacted with four lncRNAs, and the remaining mRNAs interacted with one lncRNA. Among these, there were more cis-interaction relationships than trans-interaction relations.

### GO and KEGG Enrichment Analyses

**Figure 4:**
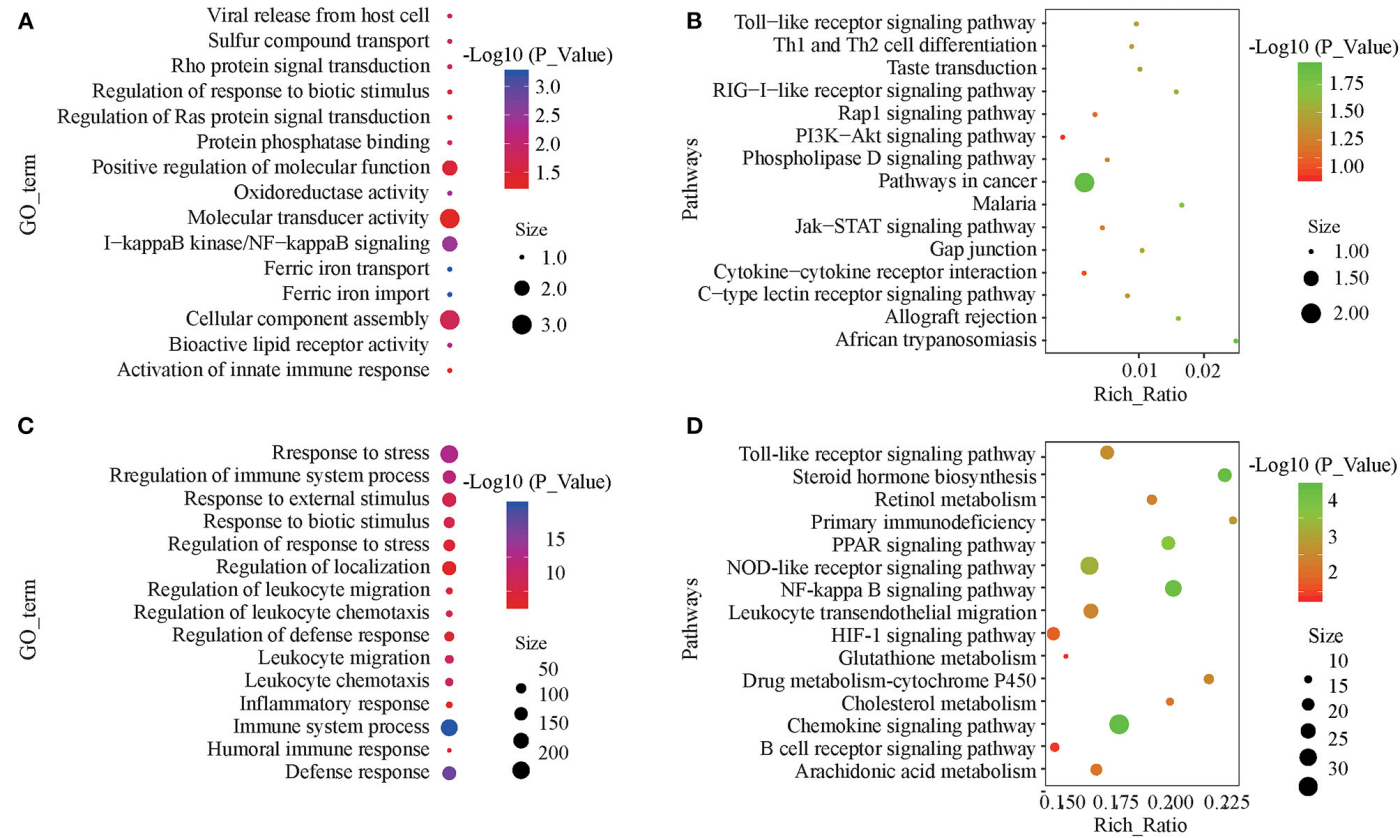
GO and KEGG enrichment analyses of differentially expressed lncRNAs and mRNAs. **(A)** Annotated GO terms of target genes of differentially expressed lncRNAs. **(B)** Enriched KEGG pathways of target genes of differentially expressed lncRNAs. **(C)** Annotated GO terms of differentially expressed mRNAs. **(D)** Enriched KEGG pathways of differentially expressed mRNAs.

The target genes of the differentially expressed lncRNAs were significantly associated with 38 GO terms, most of which were related to material metabolic processes, including ferric iron transport, trivalent inorganic cation transport, and sulfate transport (Figure 4A; Supplementary Table 4). In KEGG analysis, there were 18 significantly enriched pathways, including the RIG-I-like receptor signaling pathway, taste transduction, and the Toll-like receptor signaling pathway (Figure 4B; Supplementary Table 5). Differentially expressed mRNA was significantly enriched in 215 GO terms, most of which were related to immune function and stress response (Figure 4C; Supplementary Table 6). KEGG analysis was significantly enriched in 37 pathways, including the PPAR signaling pathway involved in fat metabolism and the NF-κβ, NOD-like receptor and Toll-like receptor signaling pathways involved in immune recognition and response (Figure 4D; Supplementary Table 7).

### qRT-PCR Validation of Differential lncRNA and mRNA Expression

**Figure 5:**
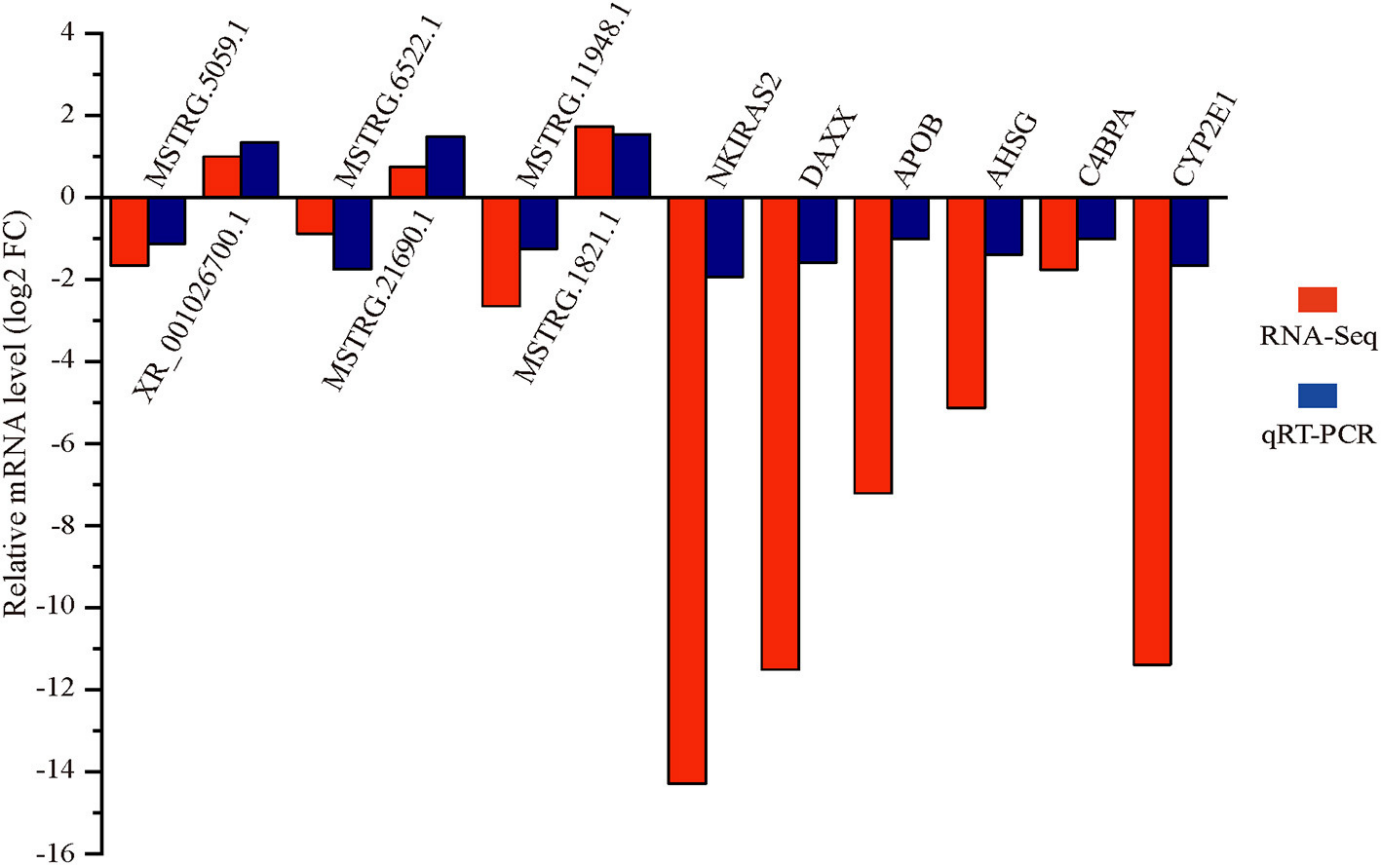
Verification of differentially expressed lncRNAs and mRNAs by qRT-PCR.

Six differentially expressed lncRNAs and mRNAs were selected for qRT-PCR verification (Figure 5). The qRT-PCR and RNA-seq results showed similar expression trends, confirming the reliability of the transcriptomic sequencing data.

## Discussion

Tibetan sheep have undergone natural selection for tens of thousands of years in the low-oxygen environment of the Qinghai-Tibet Plateau. Specifically, they have developed cardiopulmonary functions and strong low-oxygen adaptability. Under hypoxic stress, different areas of the body show different response mechanisms, forming a unique hypoxia adaptation strategy. In-depth study of the genetic mechanisms of Tibetan sheep in response to high-altitude hypoxic environmental stress can provide a theoretical basis for solving problems with animals entering Tibet, adapting to the high-altitude hypoxic environment and maintaining normal production performance in plains areas. Studying these animals can also help reveal their excellent genetic resources. Thus, we analyzed the complex life processes of Tibetan sheep and the phenotype that allows them to adapt to high-altitude hypoxic conditions at the transcriptomic level. Screening and regulating the genes related to hypoxia adaptation in Tibetan sheep will enable constructing a transcriptional expression regulatory network to analyze the genetic mechanisms of hypoxia adaptation in Tibetan sheep. GO and KEGG analyses of differentially expressed genes revealed the significant enrichment of pathways such as energy metabolism, immune response, oxidative stress, digestion and metabolism, and body temperature regulation. Strong selection on these pathways has also been performed in the study of adaptability of Tibetan pigs and yaks to high-altitude hypoxia (17–19).

Studies have shown that high-altitude hypoxic environments have an important impact on energy metabolism in animals (15, 20, 21). Similarly, we found many differentially expressed genes and signaling pathways related to glucose and lipid metabolism. Among the most significant top 20 differentially expressed genes, five were related to lipid metabolism (*PDZK1, APOB, AHSG, DAXX*, and *C4BPA*) (22–26). Additionally, in the KEGG enrichment analysis, the PPAR signaling pathway related to fat metabolism was significantly enriched. Studies on the high-altitude adaptability of Tibetan pigs have also found the significant enrichment of energy metabolism pathways. Fat is the main form of energy storage in animals' bodies. Under hypoxic conditions, the body's fat metabolism changes significantly, and the body intentionally increases its lipid oxidation and phosphorylated ATP synthesis levels with increased heat production to adapt to alpine environments (27–29). Similarly, some genes involved in fat metabolism, such as LPAR1, is also found in lncRNA target genes (30). The PPAR gene is a candidate gene for adaptation to high-altitude hypoxia. It participates in and is regulated by the HIF pathway and is closely related to the production of ATP in animals (31, 32). The activated PPAR gene can enhance fatty acid oxidation and upregulate the mitochondrial β-oxidation process. Therefore, under hypoxic conditions, Tibetan sheep can adapt to the plateau environment by increasing energy metabolism. In addition, there are certain differences in the structure of alveoli among animals inhabiting different altitudes. Type II alveoli are essential for normal lung function and regeneration after hypoxic injury. Respiration depends on surfactants produced and secreted by type II alveoli, mainly composed of phospholipids (33). Some FGF family members (*FGF11, FGF14*, and *FGF4*) were included among the differentially expressed genes. Studies have shown that FGF can stimulate the proliferation of type II alveolar cells, which in turn affects lipid homeostasis on the alveolar surface (34). This may also be caused by the enrichment of differential genes in fat metabolism.

The immune system is the body's defense barrier, and exposure to high-altitude and hypoxic environments can change the body's immune functions (35). At an altitude of 3,000 m, the number of circulating dendritic cells in human plasma is significantly reduced (36). At an altitude of 5,000 m, plasmacytoid dendritic cells in human plasma are reduced, and the TNF-α and IL-6 contents are significantly increased (37). We found that several genes, including NKIRAS2, IKBKE, and TRIM7, were involved in immunoregulation and were lncRNA target genes (38–40). Additionally, some immune-related signaling pathways, such as the NF-κβ, Toll-like receptor, and B-cell-receptor signaling pathways, were also enriched. NF-κβ is an important immune response regulator involved in innate immunity and adaptive immune responses. The differentially expressed genes identified in this study, including *NKIRAS2, IKBKE* (inhibitor of nuclear factor kappa-B kinase subunit epsilon) and *TRIM7* (tripartite motif containing 7), are key factors in the NF-κβ signaling pathway. After recognizing pathogenic microorganisms, Toll-like receptors activate NF-κβ through the MyD88 or TRIF-dependent signaling pathways, then initiate innate and adaptive immunity (41, 42). After activation, NF-κB regulates the expression of cytokines such as IL-1, IL-6, and IL-8 and releases them outside the cell to exert an early immune response effect (43, 44). This study found that the expression levels of genes such as *NF-*κ*B2, TLR2* (toll-like receptor 2), *IL1A, IL1R2*, and *IL18* were significantly lower in Tibetan sheep than in Hu sheep. The downregulation of *TLR2* expression in the lungs of Tibetan sheep may reduce the binding of *IL1A, IL1R2*, and *IL18*, thereby reducing downstream TNF-α activity (45). Decreased TNF-α activity may weaken NF-κB-induced kinase activity (NIK). Correspondingly, the decrease in NIK activity may lead to a decrease in the kinase activity inhibited by NF-κB, and the inhibition of NF-κB kinase activity leads to a decrease in the activity of the I-κB kinase (IKK) complex (46). This cascade reaction attenuates the phosphorylation of NF-κB complex inhibitor I-κB, thereby slowing its dissociation from NF-κB and reducing the level of NF-κB, which may also cause the downregulation of TNF-α expression in the lungs of Tibetan sheep (47). Our results indicate that the NF-κβ signaling pathway and its key factors play important roles in the immunoregulation of Tibetan sheep.

Animals living in high-altitude areas for a long time, under low pressure, low oxygen, low temperature and strong ultraviolet stimulation, will experience varying degrees of oxidative stress and produce large amounts of reactive oxygen and nitrogen species (48, 49). The selenoprotein, *SEPW1*, has glutathione-dependent antioxidant activity (50). *GPX1* (glutathione peroxidase-1) is a selenoprotein that can oxidize glutathione, remove excess hydroxyl free radicals and peroxides, and protect cells from oxidative stress (51). The lncRNA target gene *STEAP4* is an iron-copper oxidoreductase that uses the reducing power provided by NADPH to reduce Fe^3+^ and Cu^2+^ to Fe^2+^ and Cu^+^, which is essential for maintaining many cellular processes (52). Tibetan sheep have been living in a hypoxic environment for many years, necessitating physiological and genetic adaptation to oxidative stress. Therefore, compared with Tibetan sheep, Hu sheep are more sensitive to oxidative stress and have higher antioxidant activity. Compared with the level in Hu sheep, *GPX1* is expressed at a significantly lower level in Tibetan sheep, which is consistent with the lower GPX activity of Tibetans living on plateaus (53). In contrast, we found that the expression of *CAT*, a gene related to antioxidant enzyme activity, was significantly higher in Tibetan sheep than in Hu sheep.

Compared with Hu sheep, Tibetan sheep have always been under traditional grazing management and have a strong ability to adapt to the low-oxygen environment of the plateau. The Qinghai-Tibet Plateau is extremely deficient in forage in winter and spring, and Tibetan sheep can maintain normal reproduction even if their nutritional intake is severely insufficient, indicating that Tibetan sheep have developed unique digestive and metabolic functions. We found two related genes involved in bile acid metabolism, *SLC26A10* and *IL12A*, among the lncRNA target genes. *SLC26A10*, one of the main carriers involved in the enterohepatic circulation of bile acids, ingests plasma-bound bile salts into liver cells in a sodium-dependent manner (54, 55). *IL12A* has an immunomodulatory effect, is significantly involved in the susceptibility to primary biliary cholangitis, and can be used as a molecular target for clinical diagnoses (56, 57). Bile acid is an important component of bile. A compound with an amphiphilic molecular structure produced by cholesterol metabolism in the liver plays an important role in lipid digestion, absorption and metabolism. This suggests that Tibetan sheep can digest and absorb lipids rationally through bile acids as a defense against the harm caused by the high-altitude hypoxic environment.

Tibetan sheep have strong cold tolerance and can grow and develop normally on the Qinghai-Tibet Plateau more than 3,000 m above sea level. A series of genes involved in the regulation of body temperature were discovered in this study, such as *UCP3* (uncoupling protein 3) and *HTR4* (5-hydroxytryptamine receptor 4). Studies have shown that *UCP3* can significantly increase the oxidative respiration rate of Tibetan pig fat cells, indicating that it is an important gene in enabling Tibetan pigs to resist cold and heat (58). Another study also showed that the cold-resistance mechanism of pigs does not simply involve muscle tremor and fever, but activation of the *UCP3* protein (59). By upregulating the expression of the *UCP3* gene, Tibetan pigs promote the browning of subcutaneous white fat, increase fat production, and maintain body temperature balance. Moreover, the expression level of *UCP3* in Tibetan sheep was shown to be significantly higher than that in Hu sheep, indicating that Tibetan sheep can also mediate subcutaneous fat browning and increase the body's heat production through *UCP3*. Studies have shown that 5-HT participates in the regulation of animal body temperature (60). When an animal is in a high-temperature environment, the 5-HT neuroendocrine system is activated. 5-HT binds to 5-HTR and activates the downstream cAMP and cGMP signaling pathways, resulting in increased activity of heat-sensitive nerves and then the inhibition of heat production (61). In addition to participating in body temperature regulation, *HTR4* also plays an important role in animal gastrointestinal sensitivity and food intake (62). In contrast to the case of Hu sheep, the Qinghai-Tibet Plateau forages that Tibetan sheep live on are characterized by high fiber content, meaning that these animals have a long history of nutritional stress. These factors affect the food intake and ruminant activities of Tibetan sheep, and then affect the calories produced by metabolism to maintain body temperature stability. During this period, *HTR4* may have played an important regulatory role.

## Conclusion

Here, we systematically identified the expression profiles of lncRNA and mRNA involved in the adaptation process of Tibetan sheep to hypoxia. Functional enrichment and interaction network analysis results showed that lncRNA and mRNA may participate in the adaptation of Tibetan sheep to hypoxia via lipid metabolism. These results provide valuable resources for studying lncRNA and mRNA involved in the adaptation of animals to plateau hypoxia and can help clarify the molecular mechanisms of the adaptation of animals to plateaus.

## Data Availability Statement

The datasets presented in this study can be found in online repositories. The names of the repository/repositories and accession number(s) can be found in the article/Supplementary Material.

## Ethics Statement

The animal study was reviewed and approved by Institutional Animal Care and Use Committee of Lanzhou Institute of Husbandry and Pharmaceutical Science of Chinese Academy of Agricultural Sciences (Approval No. NKMYD201805; Approval Date: 18 October 2018).

## Author Contributions

ZL, JLiu, and BY conceived and designed the study. CY, JLi, TG, CN, and YY collected the samples. ZL performed the experiments, analyzed the data, and wrote the paper. ZL, JLi, and BY contributed to revisions of the manuscript. All authors read and approved the manuscript.

## Funding

This research work was supported by the Chinese Academy of Agricultural Sciences of Technology Innovation Project (25-LZIHPS-07 and CAAS-ZDRW202106), the National Natural Science Foundation of China (32002170), and the Special Fund of the Chinese Central Government for Basic Scientific Research Operations in Commonweal Research Institutes (1610322020004).

## Conflict of Interest

The authors declare that the research was conducted in the absence of any commercial or financial relationships that could be construed as a potential conflict of interest.

## Publisher's Note

All claims expressed in this article are solely those of the authors and do not necessarily represent those of their affiliated organizations, or those of the publisher, the editors and the reviewers. Any product that may be evaluated in this article, or claim that may be made by its manufacturer, is not guaranteed or endorsed by the publisher.
