# Supplementary material for: Comprehensive Analysis of Long Non-coding RNA and mRNA Transcriptomes Related to Hypoxia Adaptation in Tibetan Sheep
Source: Front Vet Sci. 2022 Jan 24;8:801278. doi: 10.3389/fvets.2021.801278 (PMC8818989; doi:10.3389/fvets.2021.801278)
Supplement: Supplementary file 1 [file Table_1.DOCX]

**Supplementary Table 1 Primers used in this study for qRT-PCR.**

| Gene | Primer sequences (5'-3') | Products length (bp) |
| --- | --- | --- |
| *NKIRAS2* | TCCTGGTCTACAGCACGGACA | 119 |
|  | ACACTTGTTGCCGAGGACCAC |  |
| *DAXX* | ATCTACAACTTCGGCTGTCACC | 188 |
|  | CTTCTCTTCTGTCTCTCGCTCT |  |
| *APOB* | CCTCGCCCTTGTATAACACC | 157 |
|  | ACTAACATGCCACCTTCGAT |  |
| *AHSG* | CCTTCAATGCCCAGAATAACGG | 140 |
|  | TGGATCTACGACCTCTTTAGCAA |  |
| *CYP2E1* | AAGGGCACACTCATAATTCCG | 141 |
|  | AGAAAACGCCTTGAAATGGTC |  |
| *PDZK1* | TGGCCTCCACCTTCAACCC | 120 |
|  | CCTTCTCAATCACCCGGACCA |  |
| *MSTRG.5059.1* | TTGCTTTGCGTCCAATG | 119 |
|  | TGGCCCCAACTTTGATT |  |
| *XR_001026700.1* | ATAACTATGCACATTAACGGAA | 123 |
|  | TGCACTGTTATGGATCTGTC |  |
| *MSTRG.6522.1* | CGTTGAATGTAATTGCCGAA | 125 |
|  | TCACAAAGAGTCAGACACGA |  |
| *MSTRG.21690.1* | TGGGTTACCACGCCCTC | 171 |
|  | TGGGTGTGAAGGAGTATTTCATCA |  |
| *MSTRG.11948.1* | GGTTTTCCCCAAGGCTACCAC | 105 |
|  | TGGCCGGATTCTTAACCACT |  |
| *MSTRG.1821.1* | GATTCCACATGCCTCGGAT | 101 |
|  | TCTCATTACAGTGGCTTCGTT |  |
| *ACTB* | CCAACCGTGAGAAGATGACC | 97 |
|  | CCCGAGGCGTACAGGGACAG |  |
